# Quantifying the effect of complications on patient flow, costs and surgical throughputs

**DOI:** 10.1186/s12911-016-0372-6

**Published:** 2016-10-21

**Authors:** Ahmed Almashrafi, Laura Vanderbloemen

**Affiliations:** Department of Primary Care and Public Health, School of Public Health, Imperial College London, Charing Cross Campus, Reynolds Building, St Dunstans Road, London, W6 8RP UK

**Keywords:** Complications, Discrete event simulation, Incremental LOS, Cardiothoracic surgery, Oman

## Abstract

**Background:**

Postoperative adverse events are known to increase length of stay and cost. However, research on how adverse events affect patient flow and operational performance has been relatively limited to date. Moreover, there is paucity of studies on the use of simulation in understanding the effect of complications on care processes and resources. In hospitals with scarcity of resources, postoperative complications can exert a substantial influence on hospital throughputs.

**Methods:**

This paper describes an evaluation method for assessing the effect of complications on patient flow within a cardiac surgical department. The method is illustrated by a case study where actual patient-level data are incorporated into a discrete event simulation (DES) model. The DES model uses patient data obtained from a large hospital in Oman to quantify the effect of complications on patient flow, costs and surgical throughputs. We evaluated the incremental increase in resources due to treatment of complications using Poisson regression. Several types of complications were examined such as cardiac complications, pulmonary complications, infection complications and neurological complications.

**Results:**

48 % of the patients in our dataset experienced one or more complications. The most common types of complications were ventricular arrhythmia (16 %) followed by new atrial arrhythmia (15.5 %) and prolonged ventilation longer than 24 h (12.5 %). The total number of additional days associated with infections was the highest, while cardiac complications have resulted in the lowest number of incremental days of hospital stay. Complications had a significant effect on perioperative operational performance such as surgery cancellations and waiting time. The effect was profound when complications occurred in the Cardiac Intensive Care (CICU) where a limited capacity was observed.

**Conclusions:**

The study provides evidence supporting the need to incorporate adverse events data in resource planning to improve hospital performance.

## Background

Several studies have associated postoperative complications with increased cost and Length of Stay (LOS). However, less is known about the effect of adverse events on patient flow and surgical throughputs. In hospitals with sufficient resources, complications may be less influential to overall productivity. However, when resources are constrained, complications can exert a series of sequential effects that might limit the availability of resources for other patients. To the best of our knowledge, there is no paper that has explicitly examined the relationship between complications and operational performance using simulation modelling.

An optimum bed capacity is a key factor for smoothing patient flow. However, managing beds is difficult as patients stays tend to be influenced by uncertainty. This includes occurrence of complications which trigger the use of several resources. A hospital’s efforts to manage complications is challenged by the fact that complications are difficult to predict [1]. At a certain level of capacity, a high rate of complications can substantially constrain patient flow and could reduce hospital responsiveness to urgent cases.

In many resource planning approaches, there is a tendency to focus on average utilisation of a single resource such as the operating theatre without consideration to its relationship with downstream services such as intensive care unit beds [2]. Since many hospital services are interconnected, the effect of complications should be evaluated across the patient hospital journey. Quantifying the effect of complications on patient flow permits the hospital management to evaluate key performance indicator (KPI) targets based on the existing rate of complications.

This understanding can yield several benefits such as focusing efforts on reducing certain complications and building a business case for investing in quality and safety programmes. Further, given the current economic climate, it is necessary to operate hospitals in a more efficient way. Hospitals can incur significant costs in treating complications (e.g. nosocomial infections) and might not be compensated in return.

Discrete Event Simulation (DES) has been applied to numerous health policy issues related to staffing, scheduling, and capacity management [3–5]. Much of the enthusiasm in using DES in healthcare stems from its capability to capture complexity and uncertainty. A substantial body of literature has focused on measuring patient flow improvements under alternative solutions, with the intent to provide quantitative evidence to support decisions. However, it is often that *intangible elements* such as complications tend to be ignored in DES [6]. This might be the case because it is easier to focus on measurable processes. Moreover, modellers might not have access to sensitive patient data including the details of adverse events. Because DES offers the flexibility to track interconnected and uncertain events across multiple parts of the system [7], we believe that DES is an appropriate tool for evaluating the inherent uncertainty surrounding postoperative complications and their impact on resource utilisation.

Hospital managers need to be able to evaluate efforts to reduce adverse events based on the added benefits to the patients’ health and the hospital in general. Complications that occur in the Cardiac Intensive Care Unit (CICU) might lead the care givers to allocate extra resources (e.g. more bed days). As a result, other surgeries may be cancelled due to lack of available beds. Failing to manage the ratio of beds to operating rooms (OR) results in one of the resources being underutilised [8]. Additionally, cardiac surgical patients with complications can undergo re-exploration if, for example, a postoperative haemorrhage is identified [9], potentially resulting in postponement of less urgent cases. Furthermore, patients already transferred from CICU may need to return if they experience a critical complication.

### Postoperative complications following cardiac surgery

Several factors related to patients and surgical procedures can increase the risk of complications. For example, patients with concomitant surgery (CABG and valve) are more likely to experience complications than patients with isolated surgery [10]. Patients undergoing an operation with a cardiopulmonary machine are more likely to experience an inflammatory response [11]. Blood transfusion during surgery is also associated with increased rate of morbidity [12]. The probability of complications exponentially increases as patients spend more time in the CICU [13]. On the other hand, high patient severity has been linked to occurrence of adverse events which in turn mediates on subsequent LOS [14]. For instance, Toumpoulis et al. [15] found that as severity (measured by the EuroSCORE) increases, the risk of postoperative complications tends to increase.

Cardiac post-surgical complications include some life threatening complications such as myocardial infarction. Another potentially fatal complication is postoperative bleeding which will require reoperation. Studies suggest that the reoperation rate for bleeding is in the range of 2–9 % [16, 17]. The majority of the patients will be re-operated within 24 h of the surgery. When patients experience one or more postoperative complications, their conditions can rapidly deteriorate given that most patients are above 60 years old.

## Methods

### Patients and data collection

To evaluate the effect of complications on resource use, we utilised data from 600 patients who underwent a cardiac surgery at a major referral hospital. These data were drawn from a prospectively collected database. We included all types of cardiac surgeries such as isolated Coronary Artery Bypass Surgery (CABG), isolated valve surgery, combined surgery (CABG & valve), and other types of cardiac surgery. The rationale behind this is that cardiac surgical patients, irrespective of their surgery, share the same resources (e.g. operating theatre, critical care beds, etc.) and disregarding a certain type would compromise the analysis around capacity and throughputs.

The type of collected data included demographic information, comorbidities, LOS detail, surgery detail, and postoperative complications. Several types of complications were examined such as cardiac complications, pulmonary complications, infection complications and neurological complications. In addition to the clinical data, we collected several parameters related to system operation such as surgery waiting times, non-surgical admissions, inter-arrival of elective and non-elective patients, and surgery duration. Non-surgical admissions refer to patients who are admitted for reasons other than surgery such as admission for follow-up. Elective patients are scheduled patients who are admitted based on prior appointments. Non-elective patients consist of emergency cases that require immediate care and urgent patients that are less severe than emergency cases and whom care can be delayed for few days.

### Statistical analysis

#### Regression model

To inform the simulation model building, we first examined the relationship between resource use and complications. We performed Poisson regression in order to: 1) evaluate whether complications can independently explain variation in LOS. 2) inform our simulation model building by selecting the most influential complications and 3) quantify the excess LOS and cost associated with each type of complication so they can be used in our model.

To evaluate the independent effect of complications on postoperative LOS, we adjusted the model for basic demographic characteristics, comorbidities, and type of surgery. Poisson regression has been previously found to be suitable for modelling intensive care unit and postoperative LOS data that are heavily skewed [18–20]. All analyses were two sided. A value of *P* <0.05 was considered statistically significant. Respectively, the incremental cost associated with hospital charges was estimated using the same methodology. Hospital charges were calculated based on an existing fee schedule (year 2015) for room, surgery, and investigation services (radiological and laboratory).

Excess length of stay was assessed through the marginal effect of each significant factor. Marginal Effects at the Means (MEMS) measures the changes in the response variable in relation to change in a covariate. For binary variables, the effect of discrete changes (i.e. from 0 to 1) is computed holding all other variables at their means [21]. In effect, the margins are computed for all variables related to the patient mix, the surgical characteristics and complications. Thus, they reflect the marginal changes related to the specific cohort of patients which the model was derived from. All statistical analysis was done in Stata Statistical Software: Release 12. College Station, TX: StataCorp LP.

### System description

**Fig. 1:**
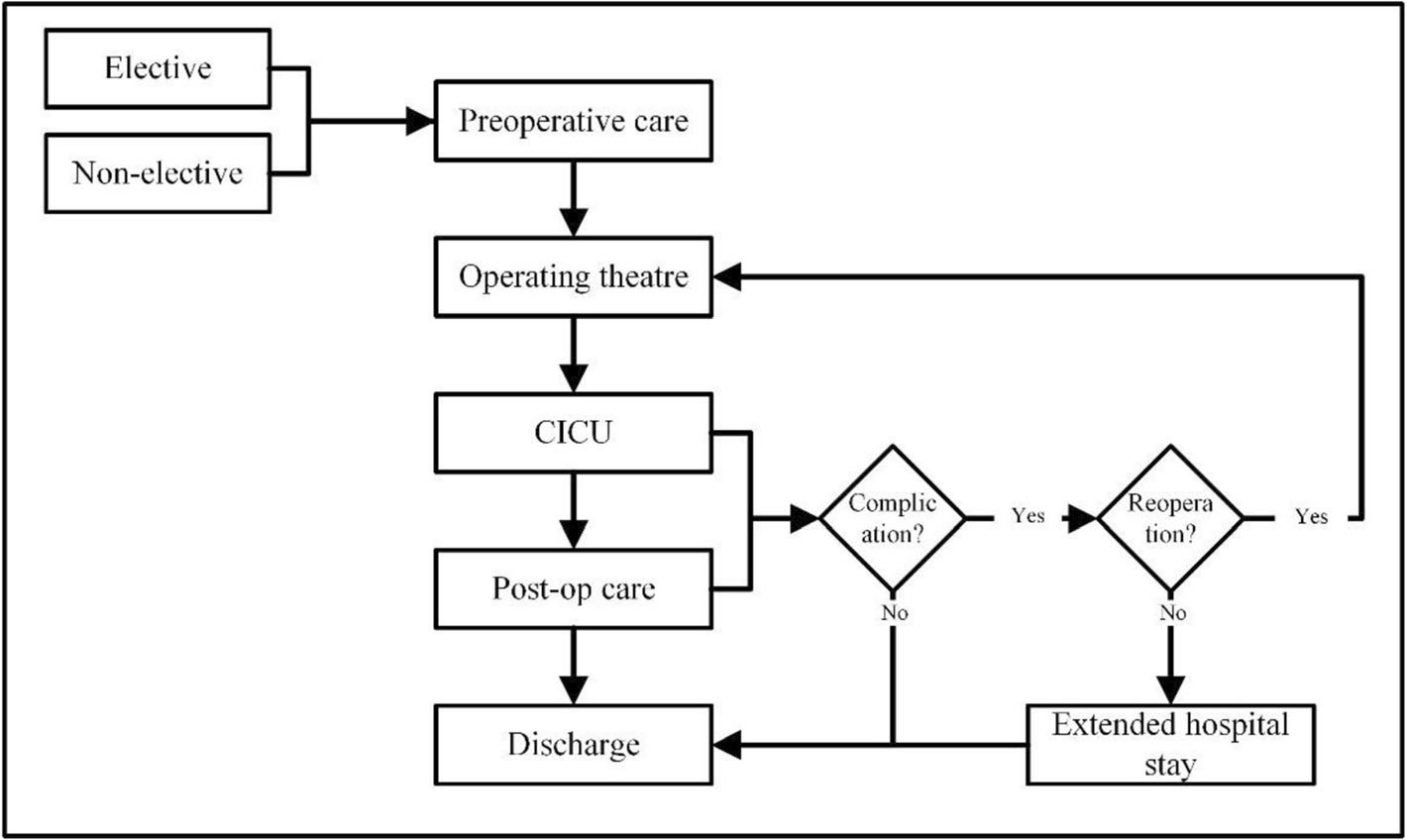
An overview of patient flow in the cardiothoracic department

The hospital under study is one of only two hospitals authorised to perform cardiac surgery in the country. Patients are referred for surgery from all other regions. Patients are also referred internally for surgery from the cardiac catheterization laboratory which is a major gateway for cardiac surgery. Following decision to operate, patients are placed in a waiting list. There is no pre-assessment clinic in the hospital which means patients have to be admitted a few days prior to their surgeries where an anaesthetist can assess their fitness for operation. Late cancellations due to unsuitability for surgery can arise, resulting in underutilisation of operating theatre time. A common surgical patient’s pathway through the system is depicted in Fig. 1. Death can occur at any stage of patient care.

There are three important components of the cardiothoracic surgical system:

1. Operating theatre: There is only a single operating theatre at the hospital that is solely dedicated to cardiovascular surgeries. Surgeries are performed 4 days a week (Sunday to Wednesday) from 8:00 am to 2:30 pm. An In-call staff can utilise the OR 24 h, 7 days a week to accommodate emergency cases which can disrupt the normal daily OR schedule. Only a single elective patient is operated per day.
2. Coronary Intensive Care Unit (CICU): This unit provides intensive care to patients immediately after surgery. Patients are kept in the CICU for at least 48 h after the surgery where they will be extubated and continuously monitored. Level of pain, vital signs, ventilation, and surgical site are carefully monitored. CICU stay is an important milestone in the patient journey. Patients who are stable can be transferred to the cardiothoracic ward to continue their recovery. Patients cannot be checked into the OR unless a CICU bed is available. The limited number of CICU beds (only five beds) have restricted OR operations in the past. The patient to nurse ratio is 1:1 in this unit.
3. The cardiothoracic ward: This is the ward where patients are initially admitted preoperatively. Some admitted patients will not be scheduled for operations for reasons such as patient refusal or unfitness for surgery. Following a surgery, operated patients who required a lesser degree of care are transferred from CICU to this ward where they will continue their recovery. For most patients the ward is the last destination before discharge. There are 18 beds available.

### Developing the DES model

**Fig. 2:**
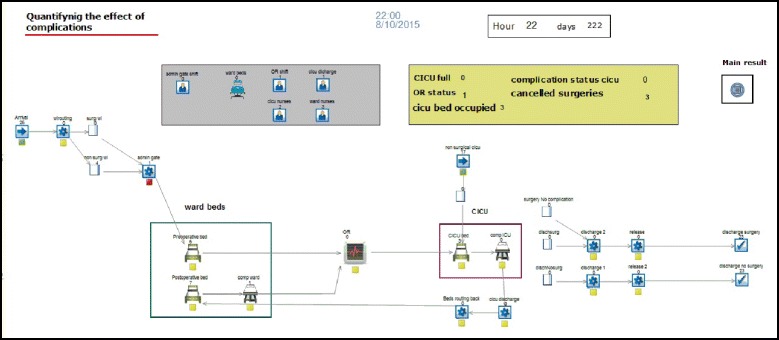
Model screenshot

We developed a DES model using SIMUL8 software package release 2015 (SIMUL8 corporation, Boston, MA). The software has its own internal language known as Visual Logic which enabled us to capture a complex representation of the real system. The model collects various statistics concerning patient types, their urgency level, duration of operation, pre and post LOS, occupancy rate, surgery cancellation, and time beds were blocked. Figure 2 provides an image of the model.

Whenever a patient enters the model, a random sample of the same type is selected from a distribution based on historical data. Type of patients included: patients with isolated CABG, isolated valve, combined CABG and valve, and other surgeries. The model then generates a profile for each type of complication based on results obtained from the Poisson regression. Once a patient is admitted, a preoperative bed will be assigned for both surgical and non-surgical patients. Preoperative LOS is determined based on distribution derived from historical data. The model then checks for CICU bed availability before selecting patients for surgery. If all beds are occupied, the model calculates the time a bed was blocked. Once a bed becomes available, priority is given to non-elective patients.

Postoperative length of stay was allowed to vary based on the type of surgery (e.g. CABG, combined surgery, isolated valve). Therefore, four types of distributions corresponding to postoperative LOS were set. From our analysis, there was an association between surgery type and postoperative LOS, sufficient to justify adding this level of detail to the model. Decisions for reoperation can be made any time post-surgery. Patients in the reoperation pathway are given priority over elective patients for surgery.

The arrival rate of elective patients in our model is well approximated by the Poisson process. It is a common approach to model arrival to a system using this type of distribution [22]. We verified this selection using the Kolmogorov-Smirnov (K-S) test. The K-S was used for fitting other distributions. The distribution that best fits the data should produce the smallest K-S values that should be below the critical K-S statistics. Inputs parameters for the model are shown in Table 1.

**Table 1:** Input parameters used to calibrate the model

Parameter	Value in baseline scenario	Distribution	Data source
Percent of admitted patients who didn’t require surgery	10 %	-	Existing data
Arrival of non-surgical patients admitted to CICU	55	Poisson	Existing data
CICU LOS	1.04, 1.6, 48, 111	Beta	Existing data
Referrals interarrival rate	33	Poisson	Existing data
Preoperative LOS (hours)	1.61, 1.3, 75, 152	Beta	Existing data
Postoperative LOS (hours):			Existing data
Isolated CABG	0.87, 1.65, 121,577	Beta	
Isolated valve	1,2.21,121,685	Beta	
CABG & Valve surgery	121, 1.48, 199	Weibull	
Other cardiac surgery	121, 1.56, 90	Gamma	
Postoperative patients returning to theatre	4 %	-	Existing data
Surgery duration (hours)	2.5,2.8,6	Triangular	Existing data

In practice, patients can experience complications during any time of their hospital stay. In the model this is governed by the same probabilities obtained from the data. Once a patient experiences a complication, the model moves that patient to the complication state. In the model, the postoperative LOS distribution was estimated based on the LOS of patients who didn’t experience complications. However, any patient who develops a complication will then be assigned an additional LOS corresponding to excess LOS that is equal to the marginal effect of the specific complication. For example, the additional LOS for a patient with pneumonia is 6.3 days, 23 days for stroke, and so on.

In order to obtain a steady state and improve output reliability, the model warm-up period and replications number were calculated. The warm-up period was determined by visually inspecting a time-series graph of surgery waiting times [7]. The value for a warm-up period was found to be approximately 6 months. The variable selected for measuring the warm-up period was the waiting time for surgery. Data were collected only after a steady state was achieved. We determined that 30 replications were required. Decision on the model scope and level of detail are referred to as simplification and abstraction [23]. In our model, it was important to include the right level of detail and system components that were directly associated with examining the problem at hand.

#### Collection of outcome measures

The effect of complications on the system operation was captured through collecting key performance indicators. In this section, we explain how these measures were derived:

1. Number of surgery cancellations: When a patient with a complication is identified in the model, a series of visual logic codes are triggered. For instance, the model inspects if a surgery was cancelled due to a complication or any other reasons as cancellations can also happen for reasons such as unavailability of theatre times or CICU beds. In the model, the following conditions must be satisfied for a cancellation to occur.
  1. All of the CICU beds are full.
  2. At least one of the patients in the CICU is having a complication.
  3. An admitted patient is ready and waiting a surgery.
  4. The operating room is available during the regular working hour.
  To distinguish between the types of surgery cancellations, the model records the number of cancellations due to unavailability of operating room sessions, unavailability of CICU bed as well as cancellations due to patients developing complications. At this stage, a patient is delayed from proceeding to the next event in the simulation. However, they will take precedence over other patients for surgery.
2. Bed turnover ratio: Bed turnover ratio is a measure of productivity of hospital beds and represents the number of patients treated per bed in a given period. It is computed according to the following formula (1).
  1 $$ \frac{Total\  number\  of\  discharges\ \left( including\  deaths\right)\ for\ a\  given\  time\  period}{Average\  bed\  count\ for\  the\  same\  period} $$
  We further calculated the “lost bed days due to complications” by observing the number of bed days that have been lost due to complication. The lost bed day rate is the forgone opportunity of admitting a new patient when a bed was not available.
3. Waiting time and waiting list: Waiting time can be a manifestation of insufficient capacity or inappropriate bed management [24]. Although complications might affect waiting time indirectly, it is important to trace their effect on waiting times to assess the hospital responsiveness. We only considered the waiting time related to patients scheduled for surgery. It should be noted that there are other elective patients who were admitted for non-surgical reasons. In the model, the order of the patient on the waiting list is updated each time a new patient enters the waiting list. At the end of the simulation, the model records both the average waiting time and the average waiting list size.
4. Surgical throughputs: We define surgical throughputs as the number of patients who successfully received operations in a given time period. This measure can be related to the surgical cancellation measure discussed previously. However, it is possible that one type of complication can lead to surgery cancellation, yet the overall surgery throughputs remain unchanged.

**Fig. 3:**
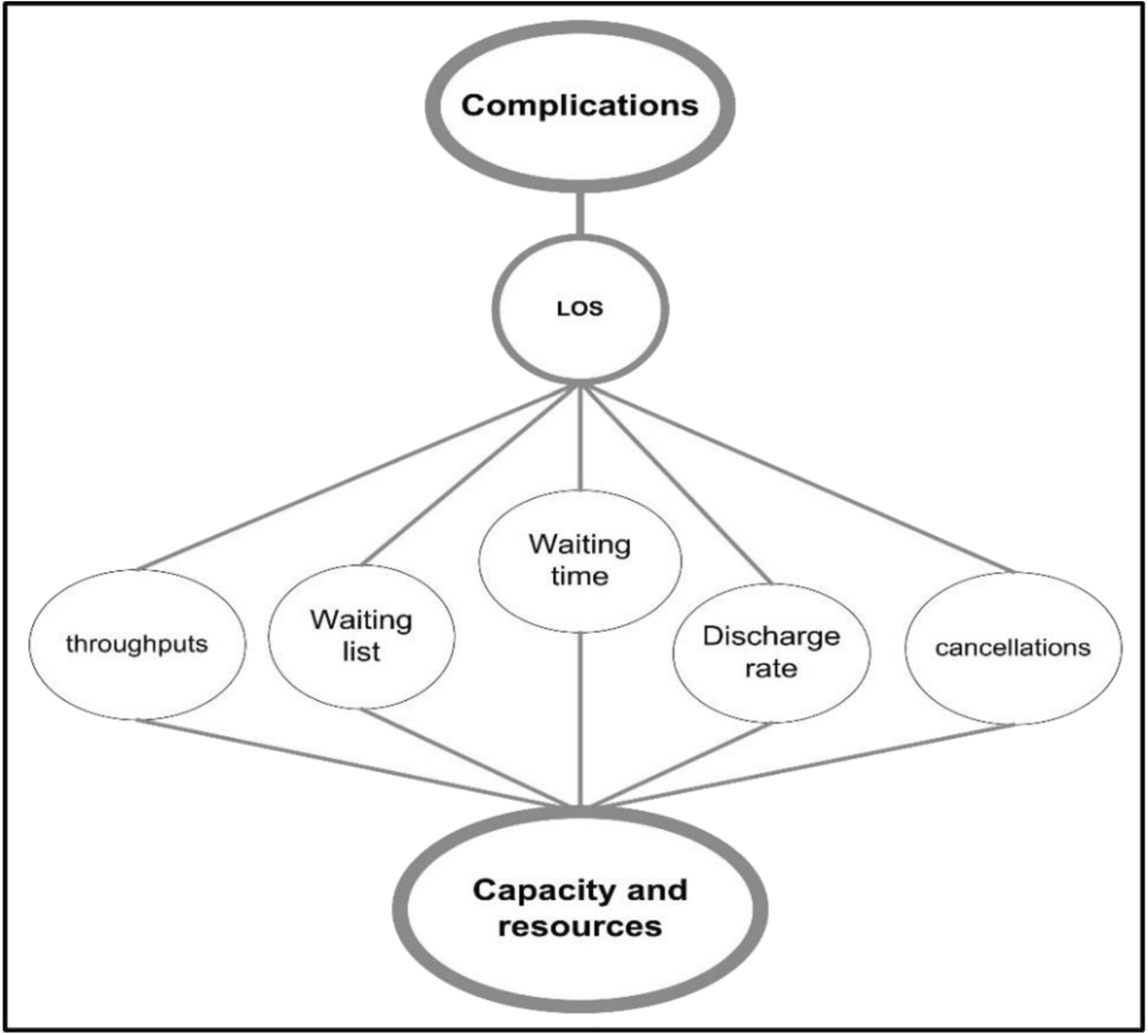
Relationships between complications, capacity and performance metrics

The previous outcome measures are also influenced by capacity and resources, as illustrated in Fig. 3, which will determine the degree to which complications can affect these measures.

### Model assumptions

Owing to unavailability of some data, we made the following assumptions to simplify the model:

- We assumed that 40 % of the postoperative complications occurred while patients were treated in the CICU unit and 60 % occurred in the ward. This assumption was made since we didn’t have relevant data regarding the location and time of where and when complications have occurred during the patient hospitalisation. However, since prolonged ventilation >24 h was more likely to occur among CICU patients, this complication was limited to the CICU stay.
- All patients were categorised as elective or non-elective. In reality, another type of ‘urgent patient’ is considered in the hospital priority system.
- Only one surgery can take place each day. Non-elective patients are given priority and are operated on the next day.

### Scenarios evaluation

We evaluated several policies that we thought might offer some potential operational improvements. These were divided into the following two categories.

- Modifying the rate of complications:
  1. An extreme scenario was assumed to eliminate all types of complications.
  2. Only complications deemed to be preventable were eliminated. In this case we focus on complications related to infections.
  3. Complications that are associated with the highest marginal hospital costs were eliminated. A marginal cost equal to or greater than the 75th percentile was used as a cut-off to indicate a high charge. This was equal to 1057.48 USD. The types of complication that met this cut-off were: permanent stroke, prolonged ventilation >24 h, other pulmonary complications, and septicaemia.
- Indirect strategies that can mitigate the effect of complications:
  4) Scheduling more surgeries by increasing the number of days when surgeries are performed.
  5) Adding more capacity to the CICU unit.
  6) Lowering ward postoperative LOS: results have shown that only 5 % of patients were discharged after the 5th postoperative day which may reflect that the LOS was influenced by local practices rather than clinical reasons.

## Results

### Results from statistical analysis

**Fig. 4:**
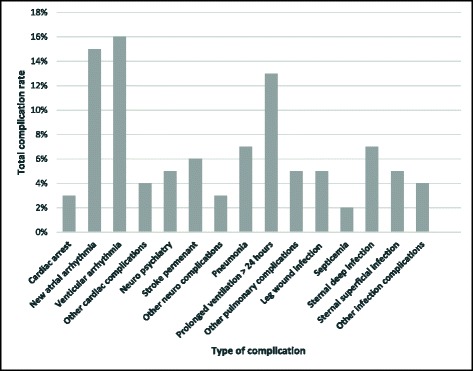
Distribution of complications among the patients who experienced complications during their hospitalisation

In our dataset, 48 % of the patients experienced one or more complications. The most common types of complications were ventricular arrhythmia (16 %) followed by new atrial arrhythmia (15.5 %), prolonged ventilation longer than 24 h (12.5 %). The distribution of complications based on type is shown in Fig. 4. Cardiac complications occurred in 26 % of the patients, pulmonary complications occurred in 17 %, neurological complications affected 9.5 %, while 16 % of the patients had infections. The underlying distribution of the postoperative LOS of patients with and without complication was statistically significant (*z* = −9.320, *P* < 0.001). On average, patients with complications spent eight more postoperative days. The median postoperative hospital LOS was 8 days.

A Kruskal-Wallis H test revealed that there was a statistically significant difference between the types of surgeries and postoperative LOS. *χ* ^2^(3) = 41, *p* < 0.001. Therefore, we further examined postoperative LOS distributions for each type individually and reflected this in the DES model.

#### The excess LOS due to complications

Table 2 lists the additional postoperative days associated with complications after adjusting for demographic variables and major comorbidities. The total number of additional days associated with infections was the highest, while cardiac complications resulted in the lowest number of incremental days of hospital stay.

**Table 2:** Marginal effect of complications on postoperative LOS

Variable^a^	Coefficient	SE	Marginal effect (days)	*P*-value
Cardiac complications				
Ventricular Arrhythmia	0.08	0.04	0.94	.025
Cardiac arrest	−0.16	0.07	−1.59	.026
New Atrial Arrhythmia	0.02	0.04	0.27	.549
Other cardiac complications	0.19	0.06	2.28	.001
Neurological complications				
Stroke permanent	1.17	0.04	22.96	< .001
Neuro psychiatry	−0.06	0.06	−0.59	.360
Other neurological complications	0.25	0.06	2.94	< .001
Pulmonary complications				
Prolonged ventilation >24 h	0.39	0.04	4.70	< .001
Pneumonia	0.49	0.05	6.33	< .001
Other pulmonary complications	0.76	0.06	11.34	< .001
Infection complications				
Sternal deep	0.35	0.05	4.30	< .001
Septicaemia	1.18	0.07	22.90	< .001
Leg wound	0.26	0.07	3.09	< .001
Sternal superficial	0.34	0.06	4.11	< .001
Other infection	0.41	0.06	5.19	< .001
Constant	1.78	.099		< .001

^a^All variables are recorded as binary (i.e. 1 for complication and 0 for absence of complication)

**Fig. 5:**
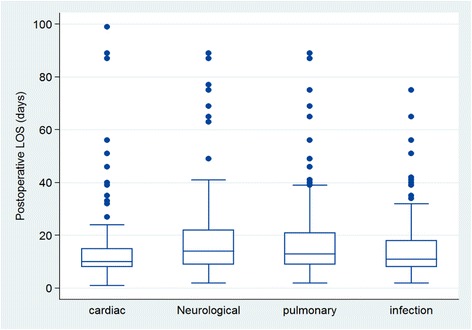
Boxplot graph for postoperative LOS distributions among patients with different complications

From Table 2, only two types of complications were not significant: neuropsychiatry complications (*p* = 0.36) and new atrial arrhythmia (*p* = .55). Surprisingly, ventricular arrhythmia which was the commonest type of complication (see Fig. 5) was associated with only one extra day of postoperative LOS. The extra postoperative LOS attributable to stroke and septicaemia (both at 23 days) was the highest. Likewise, the corresponding average change in LOS associated with pneumonia was 6 days.

The Omani Riyal was fixed to the US dollar. Thus, the total costs were converted to US dollars (USD) by a multiplication factor of 2.56, which was the existing exchange rate at the start of the study (June 2013). Cardiac surgery was associated with a sizable number of expensive complications (Table 3). The highest marginal effect for hospital charges was related to stroke (3211 USD). The extra hospital charges associated with ventricular arrhythmia was only 170 USD, despite its high prevalence. Septicaemia and other pulmonary complications had significant associated costs (2452 and 2457 respectively). On average, patients with pulmonary complications had the highest additional cost, 1415 USD, followed by 1375 USD for neurological complications, 561 USD for cardiac complications, and 793 USD for infection. The results confirmed the need to use individual complications instead of aggregating them (e.g. cardiac) as some complications were proportionally higher than others in the same category.

**Table 3:** Marginal costs associated with different types of complications

Variable	Marginal effect (US dollar)	95 % CI
Cardiac complications		
Ventricular Arrhythmia	170.01	133.94–206.11
Cardiac arrest	950.91	867.32–1034.49
New Atrial Arrhythmia	70.07	32.06–108.06
Other cardiac complications	1054.62	983.74–1125.49
Neurological complications		
Stroke permanent	3210.55	3139.09–3281.98
Neuro psychiatry	204.34	139.44–269.22
Other neurological complications	709.91	630.76–789.05
Pulmonary complications		
Prolonged ventilation >24 h	1057.48	1012.16–1102.80
Pneumonia	733.85	677.09–790.63
Other pulmonary complications	2452.22	2373.12–2531.30
Infection complications		
Sternal deep	516.02	461.28–570.78
Septicaemia	2456.99	2342.65–2571.30
Leg wound infection	598.71	531.52–665.91
Sternal superficial	169.24	106.82–231.67
Other infection	224.92	157.77–292.07

### Results from the simulation model

For each scenario, the simulation model was run for 1 year with patient waiting times, surgery cancellations, surgery throughput, bed turnover, and cost as the output of interest. Comparison of averages over multiple simulation runs was necessary to accommodate the effect of random variation (e.g. LOS duration, arrival of new patients, etc.).

A close inspection of the results revealed that patients occupying a bed due to a complication have a significant effect on several outcome measures. It was intuitive to compare the effect on the outcome measures when all complications were eliminated (scenario 1). Table 4 provides a comparison between a hypothetical state of no complications and the existing state.

**Table 4:** The effect of eliminating all complications on operational performance

Indicator	Prevalence of complication				Percentage change
	Existing state	95 % CI	None	95 % CI	
Average surgery waiting list size	23	5.85–40.39	12.33	1–25.97	−46.40
Average surgery waiting time	5 days	3.32–5.98	1.36 days	1.20–1.52	−72.80
Surgery throughputs	174	146.22–202.98	197	173.12–220.48	13.22
Surgery cancellations	9	5.81–11.52	0	-	−100
CICU bed turnover	60.76	50.84–70.68	68.21	60.02–76.41	12.30
overall bed turnover	15.66	13.13–18.20	18.23	16.06–20.40	16.41
CICU nurses utilisation	82.59 %	79.65–85.54	67.70 %	63.79–71.61	−18.03
Ward nurses utilisation	73.47 %	72.62─74.42	73.79 %	72.85─74.73	0.43

The purpose of scenario 1 (i.e. eliminating all complications), albeit unrealistic, was to estimate the burden of complications on outcome measures and provide a sense of scale of this burden. A change in all statistical indicators was observed when complications were eliminated (Table 4). For example, waiting time for surgery fell from 5 to 1.36 days, a decrease by almost 73 %. In the model with zero complications, 23 more surgeries were performed. While CICU bed turnover was improved by a reasonable number (+7.45), CICU bed turnover improved by lesser amount (+2.57). This is due to the limited number of beds in the CICU unit. The total bed days lost due to complications was 310 days. On average, each bed in the cardiothoracic department was occupied 15 days a year by patients with complications.

We further examined the effect of each type of complication on the system performance by adding each type to the model separately. Complications were aggregated based on four types (cardiac, pulmonary, infection, and neurological). Additionally, in order to estimate the effect of complications occurring in the CICU and ward separately, complications were only allowed to occur in the respective location in the model. The results are shown in Table 5.

**Table 5:** The effect of each type of postoperative complications on operation metrics based on the location where patients experienced complications

Key performance Indicator	Type of complication ^a^							
	Cardiac		Pulmonary		Infection		Neurological	
	CICU	Ward	CICU	Ward	CICU	Ward	CICU	Ward
Average surgery WT	1.37	1.39	1.53	1.74	1.57	1.48	1.61	1.51
bed turnover	18.13	17.81	14.77	17.23	19	17.51	18.90	16.64
Surgery throughputs	195	191.97	159.23	185.30	204.17	189.07	195.51	180.10
Surgery cancellations	1	0	6.31	4.83	3.17	0	5	0

^a^ The effect of each category was measured when other complications types were set at zero

As can be seen from Table 5, pulmonary complications were the most common type associated with surgery cancellations. This is the case because pulmonary complications were common in the CICU and consequently they reduced availability of beds leading to surgery cancellations. According to the model output, it was unlikely that a surgery would be cancelled if patients were treated for complications in the ward. A notable exception was when patients experienced pulmonary complications in the ward which resulted in approximately five surgery cancellations. The category “other pulmonary complications” which constitutes 4.5 % of the total type of complications was associated with substantial postoperative excess LOS (11.34 days). These complications were consequently responsible for delaying patient transfer from the CICU unit. Pulmonary complications had also reduced the surgery throughputs more than any other type of complications.

### Scenario experimentations

In this section, we provide results from other scenario experiments. Tables 6, 7, and 8 list the results from the six scenarios.

**Table 6:** The effect of the six scenarios on waiting for surgery

Scenarios	Waiting for surgery			
	Waiting time (days)	95 % CI	Waiting list size	95 % CI
Baseline	5	3.32─5.98	23	5.85─40.39
1. No complications	1.36	1.20─1.52	12.33	1─25.97
2. Eliminate infections	3.31	2.72─3.89	14.25	1─28.46
3. Eliminate high cost complications	1.711	1.49─1.93	14.06	1─28.15
4. Increasing OR operating days	3.89	3.20─4.58	15.17	1─18.92
5. Extra 1 ICU bed	3.52	2.92─4.12	2.02	1.71─2.44
6. Lowering postoperative LOS by 40 %	1.36	1.19─1.54	13.29	2─29.64

**Table 7:** The effect of the six scenarios on theatre performance

Scenarios	Theatre performance			
	Cancellations	95 % CI	Throughputs	95 % CI
Baseline	9	5.81─11.52	174	146.22─202.98
1. No complications	0	-	197	173.12─220.48
2. Eliminate infections	10	7.77─12.50	188	165.60─211.80
3. Eliminate high cost complications	3	2.26─3.74	188	164─212
4. Increasing OR operating days	15.17	11.71─18.62	204.17	186─221
5. Extra 1 ICU bed	3.87	2.87─4.87	218.47	215.80─221.13
6. Lowering postoperative LOS by 40 %	18.67	15.58─21.76	196.60	174.70─218.50

**Table 8:** The effect of the six scenarios on bed turnover

Scenarios	Bed turnover			
	CICU	95 % CI	Overall	95 % CI
Baseline	60.76	50.84─70.68	15.66	13.13─18.20
1. No complications	68.21	60.02─76.41	18.23	16.06─20.40
2. Eliminate infections	65.61	57.55─73.67	17.4	15.37─19.58
3. Eliminate high cost complications	65.33	56.92─73.73	17.50	15.28─19.73
4. Increasing OR operating days	70.58	64.50─76.66	18.45	16.87─20.02
5. Extra 1 ICU bed	75.60	75.06─76.14	19.84	19.65─20.03
6. Lowering postoperative LOS by 40 %	68.47	60.86─76.08	18.43	16.40─20.47

A substantial system improvement can be gained by lowering the rate of infections. The only outcome measure that was not improved by eliminating infections was surgery cancellation, which increased by one cancellation compared to the baseline scenario. Since septicaemia was associated with a very high incremental LOS, we examined the effect of reducing this complication by 50 %. The number of bed days that can be essentially saved by eliminating septicaemia are (23 days × the number of patients experiencing septicaemia). In the model, 50 % reduction in septicaemia resulted in reduced waiting times by 9 % from the baseline.

Scenario 3 examined the elimination of high cost complications. As such, the results compared favourably across all outcomes. The rest of the scenarios were related to modifying the existing system. An increase in OR operating days dramatically increased the number of throughputs (204 vs 174 in the baseline). However, this increase was offset by the increase in surgery cancellations (15 vs. 9 in the baseline). Additionally, waiting time improved modestly (4 days vs. 5 days). In contrast, the addition of one extra CICU bed decreased waiting list size and cancellations. It also resulted in increased surgery throughputs and bed turnover. The proportion of patients who waited for surgery fell considerably when an extra bed was added. Finally, the reduction of postoperative LOS by 40 % reduced waiting times. However, it stimulated more cancellations than any other scenario.

We attempted to estimate the variability of the results when the location of complications is changed using different assignment alternatives such as 75 %:25 % for the CICU and ward respectively. We observed large discrepancies in the model results from historical data. Therefore, we concluded that the current assignment (40 % CICU:60 % ward) of where the complications originate and are treated is the best alternative for establishing the baseline model.

In a previous paper by the first author and colleagues using the same patient dataset as the current study [25], people who died (4 % of the patient population) were found to have similar postoperative LOS distribution to those who survived. Therefore, death rate in our study has a negligible impact on resource utilisation. In hospitals where mortality is higher, the impact of death on patient flow and operational performance can be profound. Therefore, the effect of death, as a complication, cannot be overlooked in modelling patient flow and resource use.

### Model validation

There are several methods to validate a simulation model. These include comparison with historical data, face validity, input-output transformation, and sensitivity analysis among others [26, 27]. To validate our model we first met with the surgeons to ensure conceptual validity of the model (face validity). The aim was to verify that the simulation model was a credible representation of the system and the theory behind its construction was acceptable. Second, historical data from 1 year were compared against predicted data (average from 30 simulation runs) [23]. To this end, the first step was to identify the key parameters with which to validate the model. We identified two important indicators which are presented in the first column of Table 9. The *t*-test distribution was used to test the null hypothesis (there is no statistical difference between the real and simulated sets). Then the null hypothesis of the two-tailed test is to be rejected if H_0_: |T| ≤ t _α/2, n–1._ Results of this test are presented in Table 9.

**Table 9:** Validation of the model against some historical indicators using hypothesis testing. Results of 30 replications

Statistical indicator for 1 year	Observed data	Average from simulation runs	*p*-value	Variance %	H_0_
Average waiting time	11 days	9 days	0.12	−18.18 %	Accept
Completed surgeries	164	193	0.07	+17.68 %	Accept

The observed and simulated datasets were similar with small discrepancies. Thus, it can be concluded that the baseline model adequately represented the behaviour of the real world system. We could not validate the number of cancellations that occurred due to complications as there were no records kept by the hospital. However, the obtained average number of cancellations from the simulation runs was reviewed by the surgeons and found to be reasonable and approximate reality.

## Discussion

Our goal was to examine the effect of complications on some essential patient flow metrics. The findings from this study suggest that several postoperative complications were independently associated with increased hospital stay. Moreover, the marginal LOS attributable to these adverse events was a significant source for surgery cancellations, lower bed turnover rates, and extended waiting lists.

The research was motivated by the lack of an existing mechanism to measure the impact of complications on operational performance. The feasibility of modelling adverse events and their effect on hospital resources and thus operations can provide compelling evidence for quality improvement initiatives. Furthermore, given the current economic climate in Oman, it is necessary to understand how adverse events such as infections would impact bed occupancy and accessibility levels.

The use of DES modelling in this paper to assess the effect of complications on operational performance was a novel approach. The main challenge was to trace the impact of adverse events across several processes and to quantify their effect on operations. In the DES model, we integrated process characteristics such as uncertainties surrounding patient arrivals along with existing complication data. We demonstrated the utility of the DES in quantifying the effect of complications on performance measures. This modelling approach permits decision makers to understand the specific impact of a particular complication on resources (e.g. bed usage) and to provide empirical evidence on the effect on performance. Our research extends the use of DES as a methodology for operational problems involving sequential events [28] by incorporating the incremental LOS associated with complications in the patient flow.

Adverse events are directly linked to increased cost [29], and LOS [30]. The economic gain from reducing complications is well documented [31]. A study in the United States found that pneumonia following valve surgery was associated with a $29,692 increase in hospital costs and a 10.2-day increase in median LOS [32]. Post-CABG complications resulted in an incremental increase of 5.3 days in LOS among Medicare beneficiaries [29]. Patients with excessive postoperative haemorrhage were at risk of experiencing a stay in the CICU longer than 3 days, receiving ventilation longer than 24 h, and a return to the operating room for reexploration [33].

### The effect of complications on patient flow and operational performance

There is a scarcity of literature around the effect of complications on hospital performance beyond LOS and costs. However, we found that the incremental LOS associated with complications was a source of variation that affected operations. The variation was introduced as a result of a series of events triggered by complications. Much of the reduced operational performance was related to the occurrence of pulmonary complications. This can be attributable to two reasons. First, pulmonary complications such as postoperative respiratory failure are common following cardiac surgery [34–36]. This was also reflected in our dataset. For example, pneumonia and the need for prolonged ventilation were among the most commonly reported complications. Second, these complications are often associated with prolonged LOS [37, 38]. Likewise, neurological complications significantly increased waiting time and surgery cancellations. Much of this effect is related to stroke, which remains a devastating complication despite advances in perioperative care [39, 40]. Six percent of the patients in our dataset developed stroke and their LOS was among the highest of all patients.

Unlike previous studies that have found significant LOS attributable to atrial fibrillation [41, 42], the excess LOS associated with atrial fibrillation in our study was less than 7 h. Improvement in the standard treatment of this complication might have contributed toward lowering patient LOS. In general, cardiac complications had the lowest effect on waiting time, surgery throughputs and surgery cancellations. The results also demonstrated that adverse events which occurred early in the CICU had a higher impact than those that have occurred in the ward. This was due to the limited number of beds in the CICU unit. The risk factors of some of the adverse events such as stroke and pulmonary complications are known, and improvement in operational performance can be realised by effectively dealing with potentially modifiable risk factors [43, 44].

In our model, we had two waiting lists (for surgical and non-surgical patients). Surgical patients were given priority to non-surgical patients. The average waiting time for surgical patients was considerably lower as waiting time for a cardiac surgery was not an issue in this particular hospital. However, waiting for cardiac surgeries has been considered as one of the most important issues in many hospitals [45]. We incorporated waiting time in our model as many operational issues eventually manifest in the form of extended waiting times.

There are several factors that affect waiting time. Previous research has not linked them to the occurrence of adverse events. The focus has instead been on determining the effect of prolonged waiting time on morbidity and mortality [46, 47]. Under the six scenarios in this study, waiting times were compared to the existing state.

We observed that by adding an extra CICU bed, the waiting time did not improve considerably. This mainly occurred as a result of the increased number of patients. It is known that demand for resources in healthcare is dependent on supply [48]. Hence the expression ‘if you build it they will come’ can be relevant in this situation. Extra capacity can induce demand for services and unless the complication rate can be reduced, adding physical capacity might not be the optimal solution, and previous research has found that average waiting time may increase at higher levels of utilisation [49]. utilisation can be expressed by the following simple formula:

2 $$ \mathrm{utilisation}/\left(1-\mathrm{utilisation}\right). $$

For example, the utilisation of CICU beds in our example was .82. The ratio of .82/(1−.82) equates to 5.55. When an extra bed was added, this ratio increased to .86/(1−.86) = 5.85.

In the model, eliminating infections or high cost complications is a viable option that can save lives, improve patient satisfaction and contribute toward improving hospital productivity. The selection between adding more resources such as one extra CICU bed and investing in quality programmes to reduce complications should be evaluated based on how much potential cost will be avoided (e.g. costs associated with the extra LOS).

While ICU capacity strain is linked to increased morbidity and lost hospital revenue, increasing the number of ICU beds increases the hospital’s fixed costs at the same time [50]. Based on our results, some efficiency can be gained by reducing complications. This will allow the maximisation of the use of existing resources to produce the greatest output. The CICU services at the facility were in constant high demand from surgical and non-surgical patients. With a limited number of CICU beds in the country, non-refusal policy for CICU access is critical for insuring an unimpeded flow of patients.

Theoretically, most infections are preventable. In for-profit hospitals, the extra cost that might be incurred to finance quality initiatives aimed at reducing infection for example could be defrayed in part by increased revenue from the increased number of admitted patients possible by improved bed turnaround (scenarios 1, 2, and 3). However, it should be noted that high bed occupancy might leave units understaffed, and in return, increase the number of patients experiencing complications [51].

While our intention was to model postoperative complications, postoperative LOS appeared to be an issue in this hospital. Less than 5 % of the patients were discharged home after the 5th day post-surgery which could reflect the influence of local practice rather than the medical conditions of the patients. We chose to test a scenario where *postoperative* LOS was reduced by 40 %. The decrease was coupled with an increased cancellation rate. The freed capacity in the ward stimulated an increase in the number of patients who were treated in the CICU, thus contributing to the high utilisation of beds and leading to a higher cancellations rate. Respectively, *preoperative* LOS was considerably high, averaging 5 days. This has been recognised as a problem in many healthcare systems [52]. The move toward “same-day surgery” programs was a response to avoid unnecessary LOS that adds cost and might not add value to the patient’s care [53]. In general, prolonged hospitalisation is associated with an increased risk of complications [54] and may indicate shortcomings in patient safety [55].

### Limitations

#### Limitations of the statistical models

One potential limitation of this study is the extent to which its results can be generalisable. The data pertain to a specific population and specific setting, therefore, results might not be generalisable to other populations or settings with different characteristics. However, the method and interpretation of the models are generalisable.

There are various factors affecting LOS and resource utilization aside from complications, such as physician judgments, hospital policy, and adequacy of resources. The current study was limited by availability of data that were routinely collected. Therefore, the factors that were not accounted for when calculating the excess LOS attributable to each type of complication might have a significant effect. However, we think the existing data were sufficient to provide an overall measure for predicting excess LOS, as evidenced by high discriminative power.

#### Limitations of the simulation model

One of the limitations of the simulation model was the absence of data on the location where each complication originated. This can have a significant impact on results concerning resource utilisation in the CICU and the ward. As such, complications leading to prolonged LOS in the CICU would have a greater impact on patient flow than complications occurring in the ward. Second, it was difficult to track whether a surgery cancellation was due to the occurrence of complications in the downstream beds or to other reasons. Instead, we obtained subjective expert opinions to compensate for this missing variable.

The reader should be aware that the number of cardiac surgeries in the hospital under study was relatively low. The implication for this is that the pressure on resources was relatively less compared to other hospitals. Thus, the hospital might not have the incentive to expedite patient discharge. Moreover, hospitals in Oman are not required to meet specific waiting time targets for cardiac surgery. In healthcare systems where waiting times are closely monitored, LOS are expected to be shorter to accommodate more patients from the waiting list.

## Conclusions

The study provides evidence supporting the need to incorporate adverse events in resource planning to improve hospital performance. We attempted to quantify the effect of complications using DES. We found a significant impact of complications on LOS, surgery cancellations, and waiting list size. The effect on operational performance was profound when complications occurred in the CICU where a limited capacity was observed. Excess LOS spent in the hospital constitutes a lost opportunity for admitting more patients. A marked decrease in adverse events would be required to effectively deal with the negative consequences on system performance.

The growth of cardiac care services in Oman has been slow relative to the population density. Maximising existing resources would be an option as adding more resources might not guarantee a higher level of services. One way to accomplish this is by reducing avoidable complications. In our model this has not only reduced cost, but also significantly improved performance of other metrics.

As there is a scarcity in research regarding quantifying the effect of complications on patient flow and overall operational performance, we recommend further research in this area. An explicit measure should be an integral part of hospital resource planning to improve resource utilisation and perioperative patient experience. Hospitals may consider integrating the method discussed in this study into existing health information systems.

### Future work

While our study was based on cardiac surgical patients, this methodology can be applied to other specialties. For further development, researchers should aim at investigating the effect of complications related to other surgeries such as general surgeries which are associated with higher volume. Moreover, modellers should consider surgical complications that occur in the OR. In hospitals with a high demand for operating theatres, unexpected complications can lead to increased surgical time exceeding the allocated slot. This eventually results in postponement of other surgeries. Secondly, in this study we did not model the relationship between prolonged hospital stay and the increased likelihood of morbidity. Future research might consider this relationship. Finally, a hospital-wide modelling of complications is needed. A system-wide approach such as this will allow a better understanding of how complications impact resources and hospital performance.

## Abbreviations

CABG: Coronary artery bypass grafting
CICU: Cardiac intensive care unit
DES: Discrete event simulation
K-S: Kolmogorov-Simrnov test
LOS: Length of stay
MEMS: Marginal effects at the means
OR: Operating room
USD: US dollar

## References

[1] Melendez JA, Carlon VA (1998). Cardiopulmonary risk index does not predict complications after thoracic surgery. CHEST J.

[2] Azari-Rad S, Yontef A, Aleman DM, Urbach DR (2014). A simulation model for perioperative process improvement. Oper Res Health Care.

[3] Katsaliaki K, Mustafee N (2011). Applications of simulation within the healthcare context. J Oper Res Soc.

[4] Fone D, Hollinghurst S, Temple M, Round A, Lester N, Weightman A, Roberts K, Coyle E, Bevan G, Palmer S (2003). Systematic review of the use and value of computer simulation modelling in population health and health care delivery. J Public Health.

[5] Eldabi T, Paul R, Young T (2006). Simulation modelling in healthcare: reviewing legacies and investigating futures. J Oper Res Soc.

[6] Taylor K, Lane D (1998). Simulation applied to health services: opportunities for applying the system dynamics approach. J Health Serv Res Policy.

[7] Robinson S (2004). Simulation: the practice of model development and use.

[8] Bowers J (2013). Balancing operating theatre and bed capacity in a cardiothoracic centre. Health Care Manag Sci.

[9] Ranucci M, Bozzetti G, Ditta A, Cotza M, Carboni G, Ballotta A (2008). Surgical reexploration after cardiac operations: why a worse outcome?. Ann Thorac Surg.

[10] Fowler VG, O’Brien SM, Muhlbaier LH, Corey GR, Ferguson TB, Peterson ED (2005). Clinical predictors of major infections after cardiac surgery. Circulation.

[11] Butler J, Rocker GM, Westaby S (1993). Inflammatory response to cardiopulmonary bypass. Ann Thorac Surg.

[12] Al‐Khabori M, Al‐Riyami A, Mukaddirov M, Al‐Sabti H (2014). Transfusion indication predictive score: a proposed risk stratification score for perioperative red blood cell transfusion in cardiac surgery. Vox Sang.

[13] Graf K, Ott E, Vonberg R-P, Kuehn C, Haverich A, Chaberny IF (2010). Economic aspects of deep sternal wound infections. Eur J Cardiothorac Surg.

[14] Samore MH, Shen S, Greene T, Stoddard G, Sauer B, Shinogle J, Nebeker J, Harbarth S (2007). A simulation-based evaluation of methods to estimate the impact of an adverse event on hospital length of stay. Med Care.

[15] Toumpoulis IK, Anagnostopoulos CE, Swistel DG, DeRose JJ (2005). Does EuroSCORE predict length of stay and specific postoperative complications after cardiac surgery?. Eur J Cardiothorac Surg.

[16] Smárason NV, Sigurjónsson H, Hreinsson K, Arnorsson T, Gudbjartsson T (2009). Reoperation for bleeding following open heart surgery in Iceland. Laeknabladid.

[17] Kristensen KL, Rauer LJ, Mortensen PE, Kjeldsen BJ (2012). Reoperation for bleeding in cardiac surgery. Interact Cardiovasc Thorac Surg.

[18] Austin PC, Rothwell DM, Tu JV (2002). A comparison of statistical modeling strategies for analyzing length of stay after CABG surgery. Health Serv Outcome Res Methodol.

[19] Verburg IW, de Keizer NF, de Jonge E, Peek N (2014). Comparison of Regression Methods for Modeling Intensive Care Length of Stay. PLoS One.

[20] Osler TM, Rogers FB, Hosmer DW (2013). Estimated additional hospital length of stay caused by 40 individual complications in injured patients: an observational study of 204,388 patients. J Trauma Acute Care Surg.

[21] Williams R (2012). Using the margins command to estimate and interpret adjusted predictions and marginal effects. Stata J.

[22] Kelton WD, Law AM (2000). Simulation modeling and analysis.

[23] Robinson S. Conceptual Modelling for Discrete-Event Simulation. Florida: CRC Press INC; 2010.

[24] HOPE (2004). Measuring and comparing waiting lists a study in four European countries. Brussels standing committee of the hospitals of the European Union.

[25] Almashrafi A, Alsabti H, Mukaddirov M, Balan B, Aylin P (2016). Factors associated with prolonged length of stay following cardiac surgery in a major referral hospital in Oman: a retrospective observational study. BMJ Open.

[26] Nelson BL, Carson JS, Banks J. Discrete event system simulation. New Jersey: Prentice Hall; 2001.

[27] Sargent RG. Verification and validation of simulation models. In: Proceedings of the 37th conference on Winter simulation: 2005. Winter Simulation Conference; New Jersey, 2005. p. 130–43.

[28] Bayer S (2014). Simulation modelling and resource allocation in complex services. BMJ Qual Saf.

[29] Brown PP, Kugelmass AD, Cohen DJ, Reynolds MR, Culler SD, Dee AD, Simon AW (2008). The frequency and cost of complications associated with coronary artery bypass grafting surgery: results from the United States Medicare program. Ann Thorac Surg.

[30] Herwaldt LA, Cullen JJ, Scholz D, French P, Zimmerman MB, Pfaller MA, Wenzel RP, Perl TM (2006). A prospective study of outcomes, healthcare resource utilization, and costs associated with postoperative nosocomial infections. Infect Control.

[31] Eappen S, Lane BH, Rosenberg B, Lipsitz SA, Sadoff D, Matheson D, Berry WR, Lester M, Gawande AA (2013). Relationship between occurrence of surgical complications and hospital finances. JAMA.

[32] Iribarne A, Burgener JD, Hong K, Raman J, Akhter S, Easterwood R, Jeevanandam V, Russo MJ (2012). Quantifying the incremental cost of complications associated with mitral valve surgery in the United States. J Thorac Cardiovasc Surg.

[33] Christensen MC, Krapf S, Kempel A, von Heymann C (2009). Costs of excessive postoperative hemorrhage in cardiac surgery. J Thorac Cardiovasc Surg.

[34] Ji Q, Mei Y, Wang X, Feng J, Cai J, Ding W (2013). Risk factors for pulmonary complications following cardiac surgery with cardiopulmonary bypass. Int J Med Sci.

[35] Badenes R, Lozano A, Belda FJ (2015). Postoperative Pulmonary Dysfunction and Mechanical Ventilation in Cardiac Surgery. Crit Care Res Prac.

[36] Wynne R, Botti M (2004). Postoperative pulmonary dysfunction in adults after cardiac surgery with cardiopulmonary bypass: clinical significance and implications for practice. Am J Crit Care.

[37] Collins TC, Daley J, Henderson WH, Khuri SF (1999). Risk factors for prolonged length of stay after major elective surgery. Ann Surg.

[38] Lazar HL, Fitzgerald C, Gross S, Heeren T, Aldea GS, Shemin RJ (1995). Determinants of length of stay after coronary artery bypass graft surgery. Circulation.

[39] Bucerius J, Gummert JF, Borger MA, Walther T, Doll N, Onnasch JF, Metz S, Falk V, Mohr FW (2003). Stroke after cardiac surgery: a risk factor analysis of 16,184 consecutive adult patients. Ann Thorac Surg.

[40] Stamou SC, Hill PC, Dangas G, Pfister AJ, Boyce SW, Dullum MK, Bafi AS, Corso PJ (2001). Stroke after coronary artery bypass incidence, predictors, and clinical outcome. Stroke.

[41] Aranki SF, Shaw DP, Adams DH, Rizzo RJ, Couper GS, VanderVliet M, Collins JJ, Cohn LH, Burstin HR (1996). Predictors of atrial fibrillation after coronary artery surgery current trends and impact on hospital resources. Circulation.

[42] LaPar DJ, Speir AM, Crosby IK, Fonner E, Brown M, Rich JB, Quader M, Kern JA, Kron IL, Ailawadi G (2014). Postoperative atrial fibrillation significantly increases mortality, hospital readmission, and hospital costs. Ann Thorac Surg.

[43] John R, Choudhri AF, Weinberg AD, Ting W, Rose EA, Smith CR, Oz MC (2000). Multicenter review of preoperative risk factors for stroke after coronary artery bypass grafting. Ann Thorac Surg.

[44] Agostini P, Cieslik H, Rathinam S, Bishay E, Kalkat M, Rajesh P, Steyn R, Singh S, Naidu B (2010). Postoperative pulmonary complications following thoracic surgery: are there any modifiable risk factors?. Thorax.

[45] NHS. A guide to commissioning cardiac surgical services. London: 2010. http://www.scts.org/_userfiles/resources/634586925235172296_Cardiac_Surgery_Commissioning_Guide.pdf.

[46] Koomen EM, Hutten BA, Kelder JC, Redekop WK, Tijssen JG, Kingma JH (2001). Morbidity and mortality in patients waiting for coronary artery bypass surgery. Eur J Cardiothorac Surg.

[47] Sampalis J, Boukas S, Liberman M, Reid T, Dupuis G (2001). Impact of waiting time on the quality of life of patients awaiting coronary artery bypass grafting. Can Med Assoc J.

[48] Proudlove N, Boaden R (2005). Using operational information and information systems to improve in-patient flow in hospitals. J Health Organ Manag.

[49] Terwiesch C, Diwas K, Kahn JM (2011). Working with capacity limitations: operations management in critical care. Crit Care.

[50] Kahn JM (2012). The risks and rewards of expanding ICU capacity. Crit Care.

[51] Dang D, Johantgen ME, Pronovost PJ, Jenckes MW, Bass EB (2002). Postoperative complications: does intensive care unit staff nursing make a difference?. Heart Lung: J Acute Crit Care.

[52] Luigi S, Michael B, Valerie M. OECD Health Policy Studies Waiting Time Policies in the Health Sector What Works? Paris: OECD Publishing; 2013.

[53] Cella AS, Bush CA, Codignotto B (1993). Same-day admission for cardiac surgery: a benefit to patient, family, and institution. J Cardiovasc Nurs.

[54] Hassan M, Tuckman HP, Patrick RH, Kountz DS, Kohn JL (2010). Hospital length of stay and probability of acquiring infection. Int J Pharm Healthc Mark.

[55] Borghans I, Hekkert KD, den Ouden L, Cihangir S, Vesseur J, Kool RB, Westert GP (2014). Unexpectedly long hospital stays as an indicator of risk of unsafe care: an exploratory study. BMJ Open.

